# The Small-Pox Epidemic at Dewsbury

**Published:** 1904-10-22

**Authors:** 


### The Small-pox Epidemic at Dewsbury.

It has recently been stated that the Local Government Board intends to adopt drastic measures at Dewsbury, involving radical reform of management, in consequence of the continued prevalence of small-pox. We do not know what these drastic measures are to be, but the news that the authorities who are entrusted with the administration of the vaccination laws are awaking to their grave responsibility in the matter will be welcome to all right-minded citizens. It is manifest that there has been a very serious dereliction of duty on the part of somebody, and to this failure the present scourge of small-pox, with all its attendant suffering and loss of life, must be mainly attributed. The population of Dewsbury contains, or did contain previous to the present outbreak, a large proportion of persons liable to small-pox in its worst forms because they had not been vaccinated. Dewsbury a little while ago was a stronghold of the anti-vaccination agitator, and so it might be thought that conscientious objection would account for the lack of immunity among its inhabitants. But a glance at the annual reports of the Local Government Board shows that this is not so; on the contrary, unconscientious local government must be regarded as the chief cause. For, in the digest of vaccination officers' returns for 1901, it appears that of all the children whose births were registered in Dewsbury in that year no fewer than 42·3 per cent. escaped vaccination, while those exempted by conscientious objection certificates numbered only 9·3 per cent. of the total births registered. On whom, then, lies the burden of responsibility for the lives of the remaining 33 per cent. of babies who have thus been imperilled by neglect of the country's laws?

It is the duty of the guardians of every union and parish to appoint and pay one or more vaccination officers, and to assign to them districts coinciding either with a vaccination district or districts, or with a district or districts of a registrar of births and deaths. These officers must be approved by, and are subject to the jurisdiction of, the Local Government Board, from which source they are supplied with books and forms. The registrar of births and deaths must transmit once at least in every month a list of births and deaths within his district to each vaccination officer whose district wholly or partially falls within his own. Every vaccination officer must keep a book and enter minutes of the notices of vaccination given to him as above, and register the certificates transmitted to him, and each half-year he must submit a list of all cases in which certificates of vaccination have not been duly received by him during the preceding half-year to a meeting of the guardians of the union or parish in which he acts, and he must see that all children in his district are vaccinated or accounted for under the vaccination laws. From the foregoing it is evident that either the vaccination officer or the local guardians or both may be at fault, while it is equally clear that any lapse of duty of which they may be guilty must speedily come to the knowledge of their superiors, the Local Government Board. It lies, therefore, with the Local Government Board to see to it that defaulting "guardians" shall be brought to task for their bad stewardship, and that there shall be a proper observance of the administration of the laws which it is the primary duty of the Local Government Board to superintend. In the meanwhile it may safely be predicted that the task before them will not be an easy one, for nobody is more obstinate and less open to persuasion than the man who, though still obdurate, feels in his heart that he has been wrong. To use logical argument with a "guardian" who, according to a newspaper report, thinks or affirms that vaccination causes small-pox because, as the epidemic spreads, increasing numbers of people submit to vaccination, would be waste of time, and it is manifest that something more drastic than adverse comment must be used to overcome such local wisdom.

Whatever action the Local Government Board may take it is essential that it will be sufficiently energetic to ensure a proper observance of an Act which is of immeasurable benefit to the community in the security it provides for the public health and the public purse.

